# Performance Fabrics Obtained by *In Situ* Growth of Metal–Organic Frameworks in Electrospun Fibers

**DOI:** 10.1021/acsami.0c22729

**Published:** 2021-03-04

**Authors:** Maya Molco, Fabrice Laye, Enrique Samperio, Shiran Ziv Sharabani, Victor Fourman, Dov Sherman, Manuel Tsotsalas, Christof Wöll, Joerg Lahann, Amit Sitt

**Affiliations:** † School of Chemistry and the Tel-Aviv University Center for NanoScience and Nanotechnology, Tel Aviv University, Tel Aviv 6997801, Israel,; ‡ Institute of Functional Interfaces (IFG), Karlsruhe Institute of Technology (KIT), Eggenstein-Leopoldshafen 76344, Germany; § School of Mechanical Engineering, Tel-Aviv University, Tel-Aviv 6997801, Israel

**Keywords:** metal−organic frameworks, MOFs, electrospinning, polymeric fibers, value-added textiles, microreactors

## Abstract

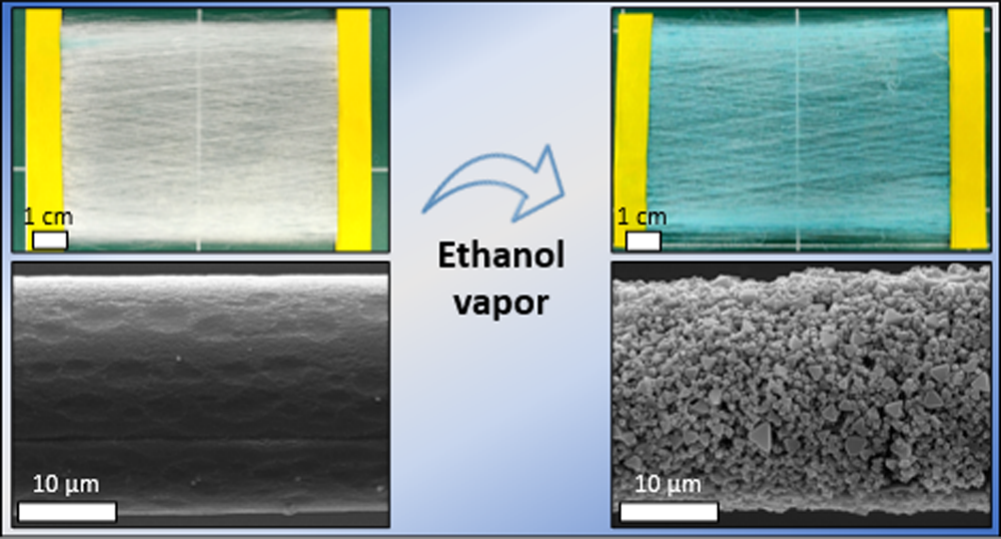

Metal–organic frameworks (MOFs) exhibit an exceptional surface area-to-volume ratio, variable pore sizes, and selective binding, and hence, there is an ongoing effort to advance their processability for broadening their utilization in different applications. In this work, we demonstrate a general scheme for fabricating freestanding MOF-embedded polymeric fibers, in which the fibers themselves act as microreactors for the *in situ* growth of the MOF crystals. The MOF-embedded fibers are obtained via a two-step process, in which, initially, polymer solutions containing the MOF precursors are electrospun to obtain microfibers, and then, the growth of MOF crystals is initiated and performed via antisolvent-induced crystallization. Using this approach, we demonstrate the fabrication of composite microfibers containing two types of MOFs: copper (II) benzene-1,3,5-tricarboxylic acid (HKUST-1) and zinc (II) 2-methylimidazole (ZIF-8). The MOF crystals grow from the fiber’s core toward its outer rims, leading to exposed MOF crystals that are well rooted within the polymer matrix. The MOF fibers obtained using this method can reach lengths of hundreds of meters and exhibit mechanical strength that allows arranging them into dense, flexible, and highly durable nonwoven meshes. We also examined the use of the MOF fiber meshes for the immobilization of the enzymes catalase and horse radish peroxidase (HRP), and the enzyme-MOF fabrics exhibit improved performance. The MOF-embedded fibers, demonstrated in this work, hold promise for different applications including separation of specific chemical species, selective catalysis, and sensing and pave the way to new MOF-containing performance fabrics and active membranes.

## Introduction

Porous solid materials with nanoscale porosity that exhibit an extremely high surface area-to-volume ratio have long been used for a variety of applications including adsorption, separation, ion exchange, and heterogeneous catalysis.^1,2^ Of this family, metal–organic frameworks (MOFs), which are made of metallic ions interconnected with organic ligands via coordination bonds, have raised a major interest because of their large surface area, the variability of the pore sizes, and the control over the chemistry of the organic ligands that enables specific and selective binding.^1−8^ In parallel to the development of new MOFs and characterization of their properties, there has been an ongoing effort for creating mesoscopic superstructures of MOFs that can enhance the MOFs’ performance and increase their processability for different applications.^9−11^ A leading approach for introducing new functional capabilities is to conjugate MOF nano- or microcrystals to other materials.^12−15^ In particular, incorporating MOFs into and onto polymeric fibers allows taking advantage of the highly developed technological capabilities of fiber processing and paves the way toward fabrication of performance fabrics and value-added textiles with a highly porous surface.^16−20^ Such fabrics hold high promise for a variety of applications, including filtering for specific chemical species, catalytic membranes, enzyme immobilization, and sensors.^21^

To date, several different methods for embedding or decorating polymeric fibers with MOFs have been reported.^22,23^ The most direct method is mixing presynthesized MOF crystals with a polymer in a melt or solution and spinning fibers of the polymer-MOF mixture.^24−30^ Another established method is the direct growth of MOFs on the surface of prespun fibers via solvothermal synthesis, sometimes utilizing a “reactive seed” approach.^23,31−35^ Such decorated fibers were utilized to a variety of applications including drug release,^36^ fabrication of antimicrobial surfaces,^37^ gas entrapment,^25,27,38^ and catalysis.^39,40^ While these approaches provide functional MOF-conjugated electrospun fibers, they may exhibit some limitations. In the case of adding presynthesized MOFs to the polymeric solution and spinning them together, most MOF crystals located inside of the fibers are not exposed to the environment. In the case of MOF growth on prespun fibers, the MOFs may be susceptible to detachment when significant mechanical loads are applied.

In this work, we demonstrate a new approach for embedding MOFs into electrospun microfibers. In this approach, the fibers contain the MOF precursors and act as microreactors that promote the *in situ* formation of MOF crystals inside of the fiber and facilitate their growth toward the outer surface of the fiber upon exposure to antisolvent vapor. Therefore, the fibers themselves template the geometry of the MOF layer. Using this *in situ* synthetic approach, we demonstrate the formation of polymeric fibers that exhibit well-exposed yet strongly embedded MOF microcrystals on their surface. The approach was established for two MOF systems: copper (II) benzene-1,3,5-tricarboxylic acid (HKUST-1)^41^ and zinc (II) 2-methylimidazole (ZIF-8).^42,43^ The resulting fibers contain more than 20% HKUST-1 and 15% ZIF-8 crystals by weight. Using this method, we obtained MOF fibers of microscale diameters that can reach lengths of several meters and utilized them for forming unwoven meshes that exhibit good mechanical properties and high durability without apparent loss of material over time. In addition, we demonstrate the immobilization of enzymes on the MOF fibers and demonstrate that the performance of the MOF-enzyme system surpasses that of fibers of similar composition without MOFs grown on their surface.

In a typical MOF synthesis, the ionic metal centers react with the organic multidentate ligands to form a network via coordination bonds. The obtained products and the reaction rate are strongly affected by the selected precursors and by the parameters of the synthesis including the solvent, temperature, pressure, compositions, and additives. Thus, the approaches for MOF synthesis are highly diverse and can vary significantly for different MOFs.^44,45^ Synthesizing MOFs within a polymeric matrix has several key hindrances that must be overcome. First, to achieve a homogeneous distribution of the precursors, the selected solvent must dissolve a sufficient amount of both the precursors and the polymer. Second, the precursors’ concentration must be high enough to enable the growth of the MOF crystals, which results in a solution with a relatively high ionic strength. Many polymers tend to salt out of solution at such high ionic concentrations, and thus, a polymer that stabilizes the salt and does not salt out is required as a scaffold polymer. Third, as the MOF growth process occurs post-fabrication, there should exist an antisolvent that can penetrate the polymer matrix and induce the formation of the MOF crystals inside it. Last, the MOF precursors must be able to transport through the polymer matrix to maintain the growth of the crystals as they form.

**Figure 1:**
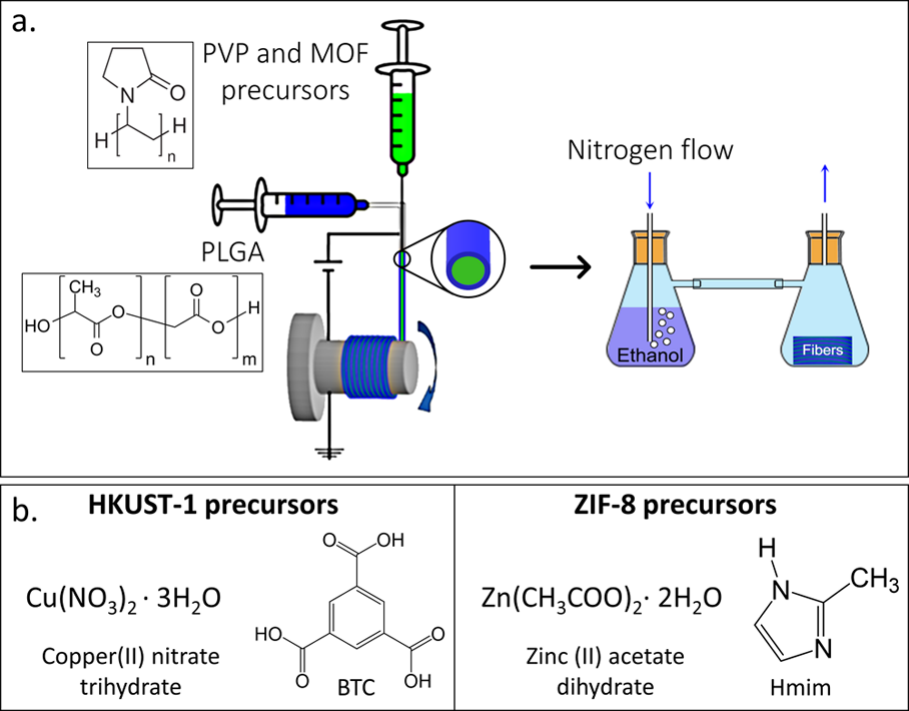
(a) Schematic illustration of the core–shell architecture for the fabrication of the MOF fiber mesh. The core solution (in green) contains PVP + MOF precursors (Cu(NO_3_)_2_·3H_2_O and BTC for HKUST-1; Zn(O_2_CCH_3_)_2_·2H_2_O and Hmim for ZIF-8), and the shell solution (in blue) contains PLGA. The two solutions are injected simultaneously to achieve a core–shell structure (left). After the fabrication of the fibers, they are flushed with ethanol vapor, inducing the growth of the MOF crystals (right). (b) Molecular structures of the precursors of the two MOFs: HKUST-1 precursors (left) and ZIF-8 precursors (right).

As a proof of concept for the feasibility of the method, two MOFs were chosen: HKUST-1, which is composed of copper (II) ions and benzene-1,3,5-tricarboxylic acid (BTC), and ZIF-8, which is a member of the zeolitic imidazolate framework (ZIF) family and is composed of zinc (II) ions and 2-methylimidazole. The growth of the HKUST-1 and ZIF-8 fibers was done in a two-step process, depicted schematically in Figure 1a. In the first step, a core–shell fiber, which contains the MOF precursors, was obtained using electrospinning.^46^ In a typical procedure, the MOF precursors that consist of a metal salt and an organic linker were dissolved together with polyvinylpyrrolidone (PVP) in dimethyl sulfoxide (DMSO). PVP was chosen as the carrier polymer because it chelates the metal ions, anchoring them into the backbone of the fiber, and hence assures a homogeneous distribution of the precursors throughout the fiber.^47−49^ In addition, the PVP acts as a thickening agent that increases the viscosity of the core solution and stabilizes the electrospinning. Despite the stabilization induced by the PVP, obtaining a stable spinning of PVP loaded with the precursors is hard to maintain due to the high content of free ions in the solution. Electrospinning of the system in a core–shell architecture with a solution of polylactic-*co*-glycolic acid (PLGA) as a shell resulted in a significant stabilization of the jetting process and in the formation of uniform and continuous fibers. This architecture is obtained by dispensing the core and shell solutions simultaneously through a metallic needle composed of two coaxial needles, one inside the other. Due to the laminar flow of the polymer solutions, the core–shell morphology dictated by the needles’ architecture is kept within the fiber. In the second step, the obtained fibers are exposed to ethanol vapor. The ethanol vapor penetrates the polymer fibers and acts as an antisolvent for the MOF precursors, leading to the *in situ* crystallization and growth of the MOFs toward the surface of the fiber. A similar approach was demonstrated before by Ameloot *et al*.^50^ and Cravillon *et al.*(51) for the growth of MOFs in the liquid phase and the gas phase;^50−52^ however, to the best of our knowledge, it is the first time the method is deployed for the growth of MOFs in a polymer matrix.

**Figure 2:**
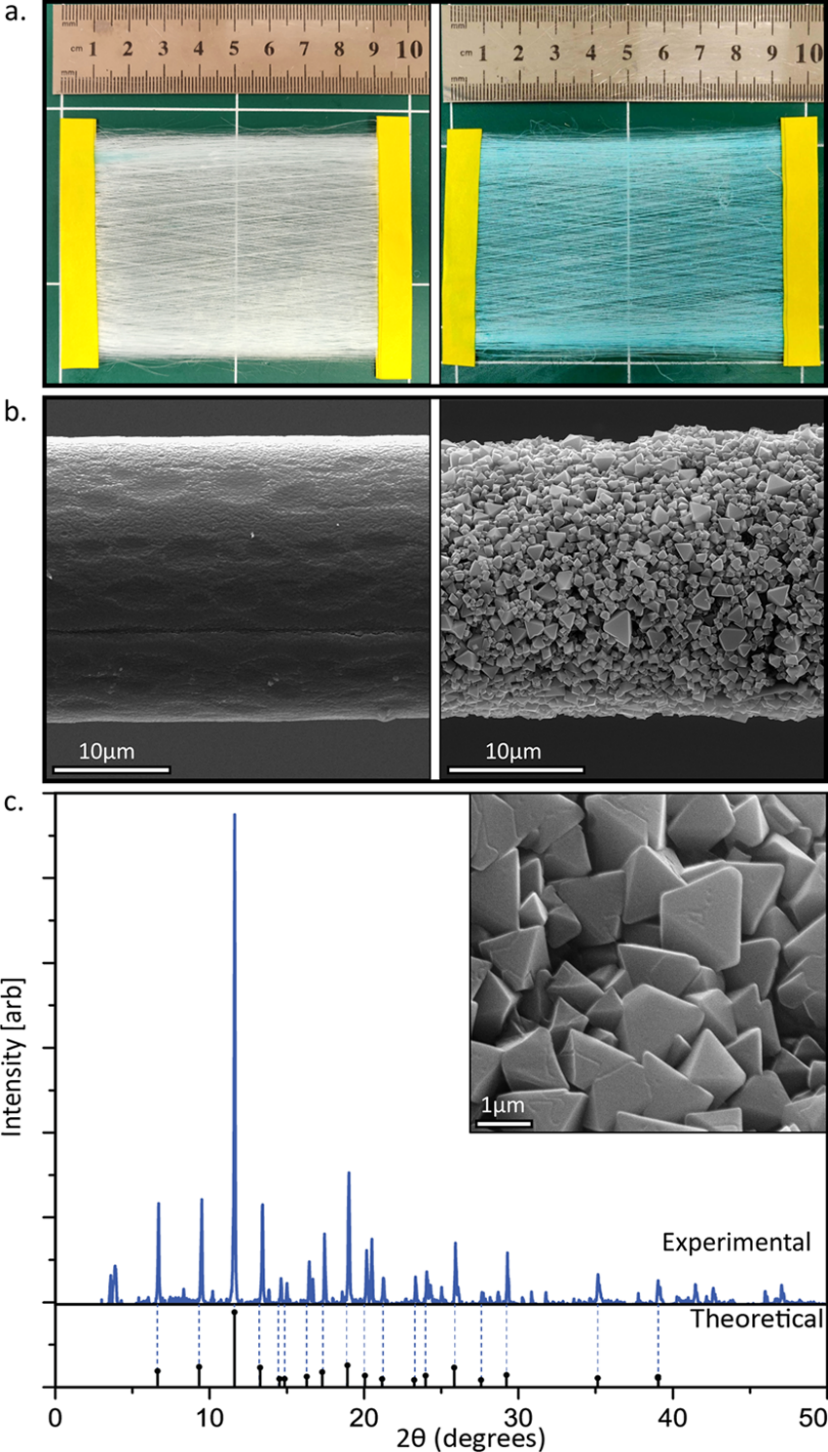
(a) Electrospun mesh of fibers containing HKUST-1 precursors before (left) and after (right) exposure to ethanol. (b) The growth of the HKUST-1 crystals is clearly seen when comparing a single fiber before (left) and after (right) exposure to ethanol. (c) Obtained XRD pattern (top) in comparison to the theoretical pattern for HKUST-1 (bottom). The inset shows a zoom-in of the obtained HKUST-1 crystals.

Figure 2a shows a mesh made of the HKUST-1 fibers before (left) and after (right) exposure to ethanol vapor. Upon exposure to the vapor, a significant change in the fibers’ color from pale to dark cyan is observed. Scanning electron microscopy (SEM) micrographs of single fibers indicate that before exposure to ethanol vapor, the fiber is relatively smooth (Figure 2b, left). After exposure to ethanol vapor (Figure 2b, right), a dense layer of microcrystalline material emerges on the surface of the fiber. The obtained crystals are well embedded in the polymer fibers, indicating that the crystal growth started within the polymer matrix and advanced toward the surface. Size distribution measurements based on SEM micrographs indicate an average fiber diameter of 19 ± 4 μm (Figure S1a). The MOF crystal average diameter is 0.94 ± 0.31 μm (Figure S1b) and they exhibit octahedral morphology, typical of HKUST-1.

Further identification of the crystal structure was obtained using X-ray diffraction (XRD). Figure 2c shows the XRD pattern of a sample of fibers after exposure to ethanol vapor (blue, solid line). The positions of these peaks correspond well with the positions of the theoretical peaks obtained from the theoretical model of HKUST-1 (black peaks, lower panel). However, a few small additional peaks that could not be identified are apparent and might indicate the existence of small contamination in the sample.

To analyze the yield of the MOF growth process, we utilized several different approaches to quantify the mass percentage of the MOFs in the fibers. Thermogravimetric analysis (TGA) of the HKUST-1 fibers indicated that the fibers contain ∼9% copper by weight (Figure S3a). The total amount of copper added to the solution is 9%, indicating that the amount of copper in the fibers is preserved at the jetting stage. However, this TGA cannot distinguish between unreacted copper and copper in the MOF crystals.

Quantitative analysis of the ratio of crystalline to amorphous phases from the XRD measurement for the HKUST-1 fibers indicated 23% crystalline material by weight. Assuming that the polymer phase is almost completely amorphous, the crystalline phase can be predominantly attributed to HKUST-1. Direct gravimetric analysis of the HKUST-1 content was performed by weighing a mesh of HKUST-1 fibers and then dipping it in a mixture of methanol and chloroform (1:1 in volume). The solution completely dissolved only the precursors and polymers (PVP and PLGA), leaving behind the undissolved HKUST-1 crystals. The crystals were washed and centrifuged several times to remove any residues of polymers and unreacted precursors. The remaining pellet containing only the HKUST-1 crystals was dried and weighed. The process was performed on four different samples, yielding an HKUST-1 weight percentage of 22 ± 8%, in consistency with the XRD analysis.

According to the initial amounts of copper and BTC that were loaded in the system, in the case that all the precursors reacted to form HKUST-1 MOF, the content of the MOF in the sample should be 45% by weight. According to the above-mentioned analysis obtained using two different techniques, we obtained that the fibers consist of ∼22% HKUST-1 by weight; thus, the yield of the synthesis is approximately 50%.

**Figure 3:**
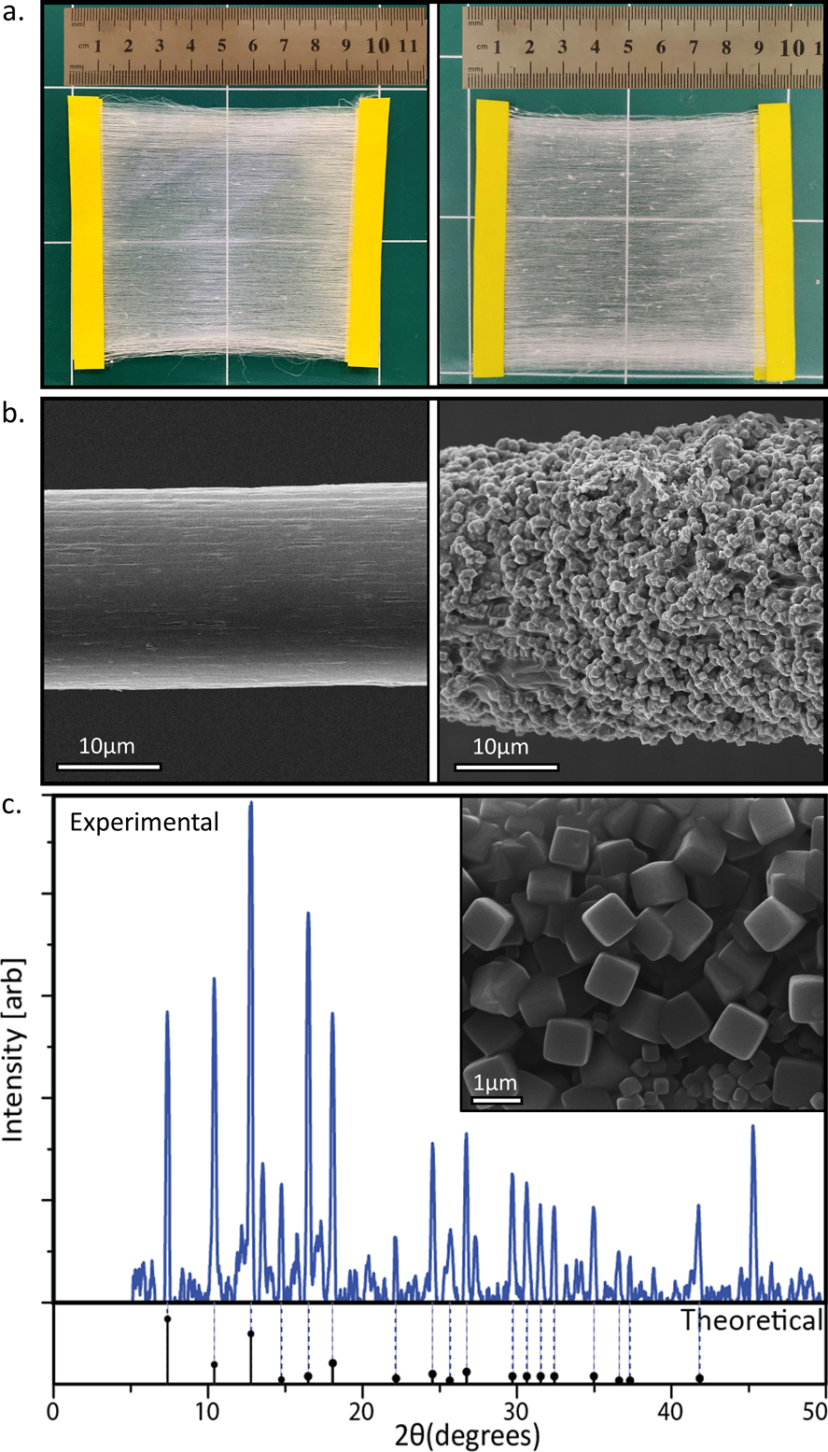
(a) Electrospun mesh of fibers containing ZIF-8 precursors before (left) and after (right) exposure to ethanol. (b) The growth of the ZIF-8 crystals is clearly seen when comparing a single fiber before (left) and after (right) exposure to ethanol. (c) Obtained XRD pattern (top) in comparison to the theoretical pattern for ZIF-8 (bottom). The inset shows a zoom-in of the obtained ZIF-8 crystals.

A similar fabrication scheme was used to jet fibers and meshes embedded with ZIF-8 precursors, as depicted in Figure 3a. TGA measurement indicated a weight percentage of 10% zinc in the sample, in accordance with the initial amounts of zinc in the solution (Figure S3b). SEM micrographs indicate the formation of microcrystals that cover the surface of the fiber upon exposure to ethanol vapor (Figure 3b). Size distribution measurements indicate a fiber average diameter of 17 ± 3 μm (Figure S1c) and an MOF crystal average diameter of 0.84 ± 0.32 μm (Figure S1d). Figure 3c depicts the XRD pattern of the sample after exposure to ethanol vapor. The diffraction pattern obtained from the fibers (blue, solid line) fits well the theoretically calculated pattern of ZIF-8 (black, lower panel), indicating the existence of ZIF-8 MOFs in the fibers. Again, a few small additional peaks that could not be identified appeared in the spectrum, which can point toward the existence of a small amount of contamination in the sample.

Quantitative analysis of the ratio of crystalline to amorphous phases from the XRD measurement for the ZIF-8 fibers gave a value of 15% crystalline material by weight. This value is consistent with the gravimetric analysis of the ZIF-8 content, which yielded a weight percentage of 18%. The precursors added to this system were 49% by weight; therefore, ∼15% of ZIF-8 crystals on the fibers give a yield of ∼31%. We attribute the incomplete consumption of the precursors to the anchoring of the copper or zinc ions to the PVP backbone, which reduces their availability for reacting and composing the MOFs.^56^ The summary of the crystal percentages in both systems is presented in section S3 in the Supporting Information.

**Figure 4:**
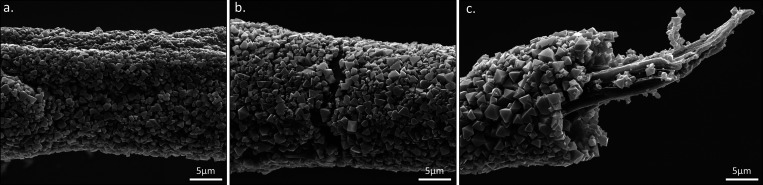
Upon stretching of an HKUST-1 embedded fiber, initially, the polymeric fiber extends (a), leading to the formation of fractures in the external crystal layer (b). Eventually, the polymer fiber backbone is torn, revealing an embedded crystal shell wrapping the polymeric fiber backbone (c).

In both HKUST-1 and ZIF-8 MOF fibers described above, the fibers are smooth after the jetting, and only after exposure of the fibers to ethanol vapor, MOF crystals emerge and appear on the surface of the fibers (Figures 2b and 3b). For both systems, the formed crystals are well embedded in the fibers, indicating that the crystals grew from the inside toward the outer surface of the fibers. This is further emphasized when inspecting fibers that were mechanically pulled in the scanning electron microscope. Figure 4 shows a micrograph of an HKUST-1 composite fiber that was pulled after exposure to ethanol and the growth of the crystals. Before pulling, the outer MOF layer is intact and covers the entire fiber (Figure 4a). After pulling, some fibers exhibit fractures in the MOF layer, exposing the polymer fiber smooth backbone (Figure 4b). Further pulling of the fibers led to stretching of the polymer backbone until it tore down (Figure 4c). Inspecting the torn fibers in the scanning electron microscope clearly shows that the MOF crystals form a dense shell around the fiber backbone, which is kept intact even upon the rupturing of the fiber. Furthermore, there is no evidence for the formation of crystals inside the fiber itself. Similar indication was obtained also in sectioned fibers of HKUST-1 (Figure S4) and for ZIF-8 fibers that were mechanically pulled (Figure S5).

To further elucidate the growth process, the elemental content of the surface of HKUST-1 composite fibers was examined by X-ray photoelectron spectroscopy (XPS) before and after exposure to ethanol vapor. The XPS measurement indicates the average elemental and chemical composition of the fibers’ surface up to a thickness of ∼10 nm and can give direct information regarding the changes in the metal content throughout the process.

**Figure 5:**
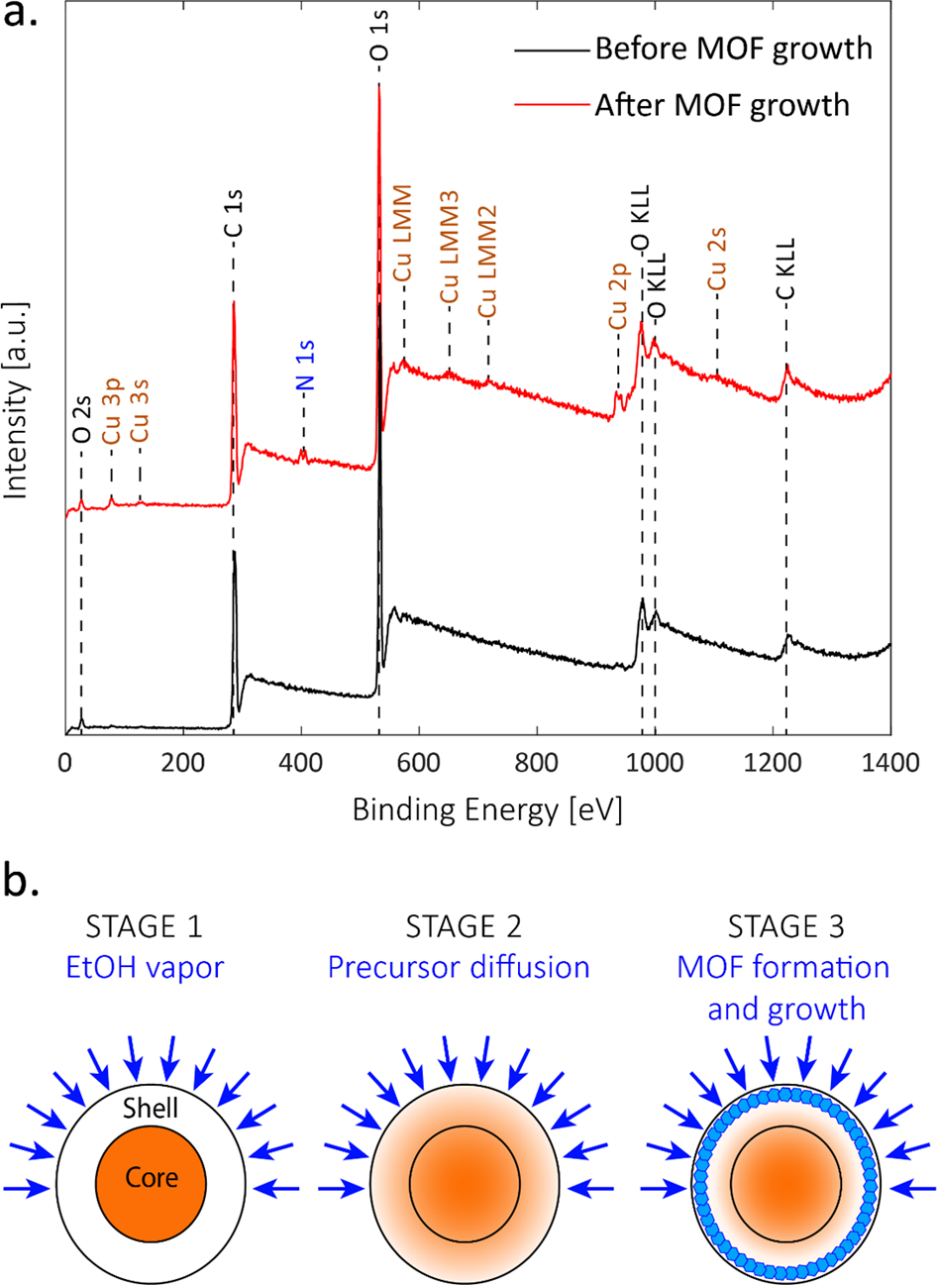
(a) XPS spectra of the fibers before (black) and after (red) exposure to ethanol vapor. The peak assignment is reported above the peaks, indicating the emergence of copper and nitrogen on the surface after the exposure. (b) Schematic illustration of the proposed growth scheme. At the first stage, the precursors are positioned only at the core. As the ethanol vapor penetrates the fiber, the precursors diffuse toward the surface (stage 2), and once the ethanol concentration increases enough in the fibers, the growth process begins, and MOF crystals are formed on the surface of the fibers (stage 3).

Figure 5a shows the XPS spectra of the fibers before (black) and after exposure to ethanol vapor (red). The assignment of the peaks is reported in the graph. In both spectra, strong peaks for oxygen and carbon are detected and can be attributed to the polymers. However, upon exposure to ethanol, additional peaks, which correspond to copper and nitrogen, emerge. The only sources of nitrogen in the fibers are the PVP and the copper salt, and hence, these results indicate that, initially, there are almost no copper atoms and no PVP on the surface of the fibers. This is indeed expected because both materials reside in the core of the fiber. However, upon exposure to the vapor, migration of both copper and PVP from the core toward the surface of the fibers is initiated. This is further emphasized when examining the amounts of atoms extracted from the XPS measurements summarized in Table 1. The concentration of copper on the surface rises from ∼0 to 6% mass, which is close to the average weight percentage of copper in the entire fiber, as calculated according to the jetting parameters.

**Table 1:** Elemental Surface Weight Percent of the Fiber before and after Exposure to Ethanol Compared to the Weight Percent of the Atoms (Excluding Hydrogen) in the Fiber Based on the Jetting Parameters

	surface weight % before exposure	surface weight % after exposure	weight % in the entire fiber
copper	0.93	6.04	10.21
nitrogen	0.19	2.30	6.41
carbon	51.83	48.46	38.98
oxygen	47.05	43.20	44.41

Based on these results, we propose a possible growth scheme that consists of two processes that occur simultaneously upon exposure to ethanol. On the one hand, the ethanol vapor penetrates the polymer matrix and leads to its plasticization. This promotes the diffusion of the PVP and the MOF precursors toward the fiber edges along the concentration gradient.^53−55^ On the other hand, ethanol is an antisolvent for the MOF precursors, and thus, once the concentrations of ethanol and the precursors increase the formation and growth of the MOF crystals occurs. The exact position of the crystal growth depends on both the concentration gradients and diffusion rates of the ethanol and the precursors across the fiber, but based on the obtained results, it occurs close to the surface of the fiber, and the precursors migrate rapidly so that the significant growth of the crystals occurs from the surface outward.

**Figure 6:**
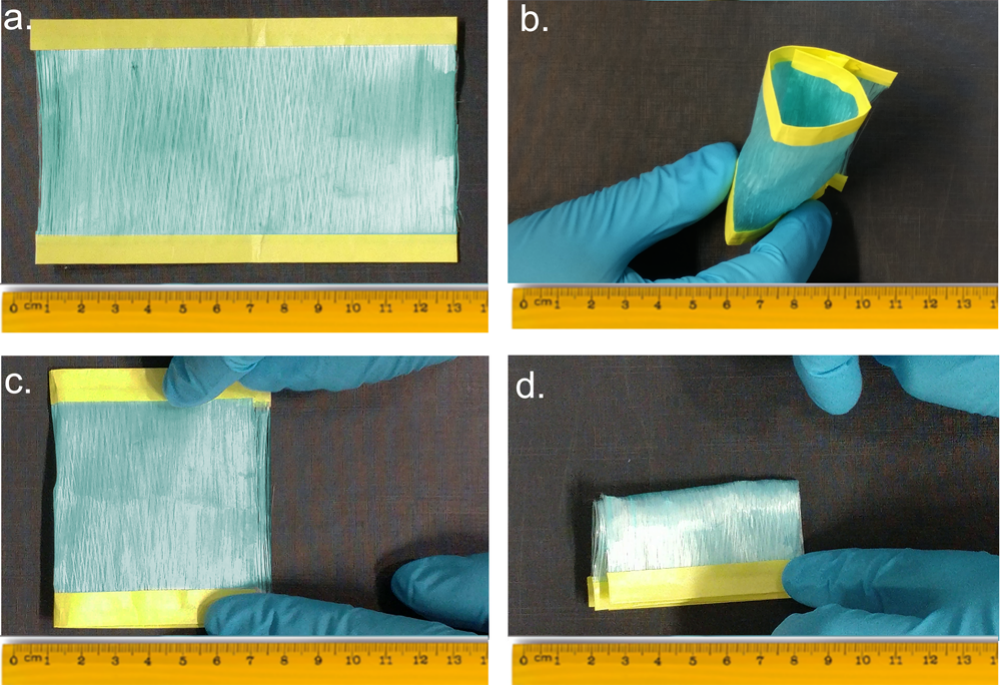
(a) A 12 cm × 7 cm nonwoven mesh fabric of HKUST-1 fibers after exposure to ethanol vapor can go through (b) rolling and (c, d) folding without tearing of the fibers or apparent release of the embedded MOF crystals.

A key aspect of utilizing such systems for different applications is the mechanical strength of the obtained fibers and of the nonwoven fabrics formed from them. Figure 6a shows a representative example of a 12 cm × 7 cm mesh made of HKUST-1 composite fibers that were spun back and forth over a rotating drum, followed by exposure to ethanol vapor. As shown in Movie S1 and Figure 6b–d, the obtained mesh can be skewed, stretched, rolled, and folded without tearing the mesh or the fibers and without the release of any of the embedded MOF crystals. The mechanical strength of the MOF layer is also observed in the single fiber level, and mechanical deformation of the single fiber did not yield detachment of the crystals (Figure S6 and section S6).

**Figure 7:**
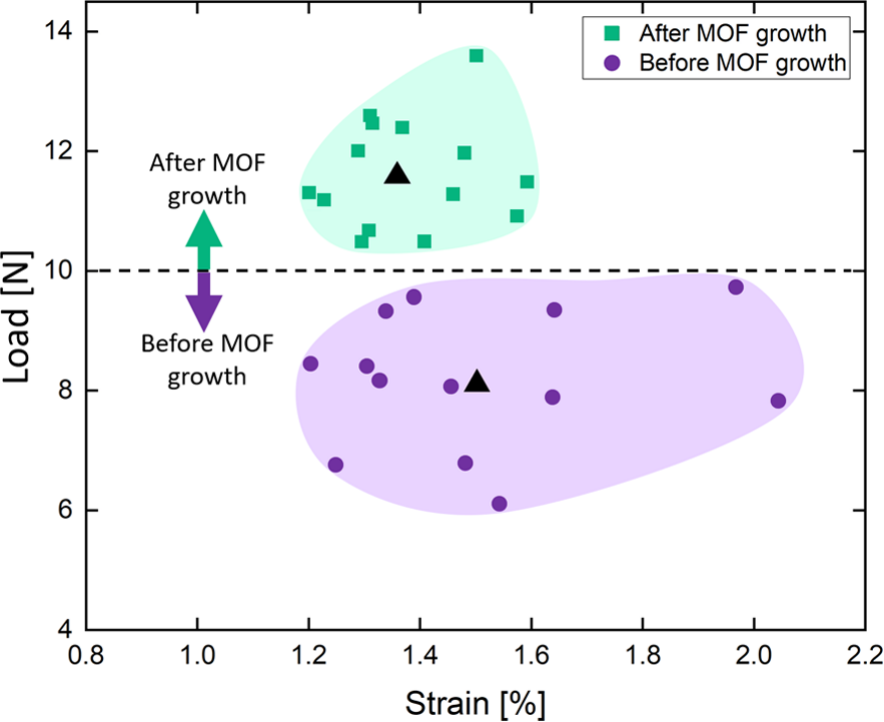
Tensile strength for HKUST-1 fabrics before (purple) and after (green) exposure to ethanol vapor. Average value for each set of samples is depicted as a black triangle marker in the center of mass of each set. Ellipsoids are guidance to the eye.

To examine the effect of the MOF growth on the mechanical properties of the fibers, tensile strength measurements were performed on nonwoven HKUST-1 fabrics before and after exposure to ethanol vapor. Figure 7 shows the tensile strength distribution graph of 28 HKUST-1 fabric samples of similar dimensions and fiber densities: 14 before exposure to ethanol vapor (untreated fabrics) and 14 HKUST-1 fabric samples after exposure to ethanol vapor (treated fabrics). While all the untreated fabrics exhibit a tensile strength below 10 N, all the fabric samples after the growth of the MOF crystals exhibit a clear increase in the tensile strength to above 10 N. Weibull statistical analysis performed on the results indicates a significant statistical difference between the two sample types (Figure S9), with average tensile loads of 8.17 ± 1.10 and 11.63 ± 0.91 N for the untreated and treated samples, respectively, and a Weibull modulus of 6.07 compares to 13.92, respectively, indicating the durability of the latter. These significant differences indicate that the crystals enhance the mechanical strength of the fibers and decrease the amount of defects in the fibers, possibly by forming a strong interaction with the polymer matrix. Full description of the tensile measurements, including the Weibull statistical formulations, the Young modulus, and the distributions, is provided in section S7 in the Supporting Information.

In recent years, MOFs have been proven to be highly potent solid scaffolds for the immobilization and stabilization of enzymes,^56−59^ and the immobilization of a variety of enzymes on different MOFs was demonstrated.^59−63^ The MOF composite fibers offer a unique combination of both strong and protective support for the enzymes and the flexibility and ease of handling of the fabrics and open the path for the fabrication of active catalytic membranes for industrial and environmental applications.

To illustrate the potential of the MOF fibers as scaffolds for enzyme immobilization, we chose to examine the immobilization of the enzyme horseradish peroxidase (HRP) on the MOF fibers.^59,63,64^ HRP is a metalloenzyme that utilizes hydrogen peroxide to catalyze the oxidation of different organic substrates. In particular, HRP catalyzes the oxidation of fluorogenic substrates that form a fluorescent product, which allows following the reaction kinetics through the fluorescence intensity of the product.

**Figure 8:**
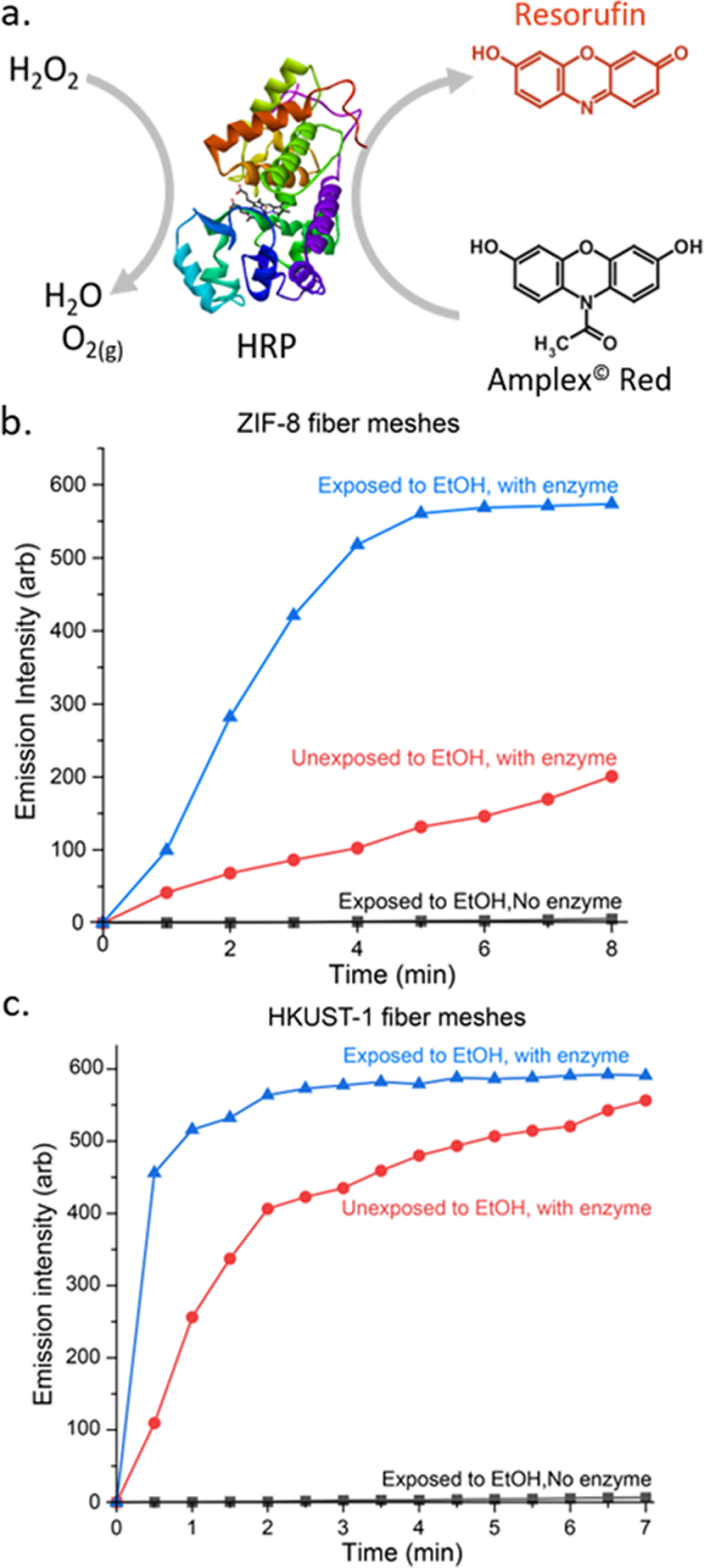
(a) Catalysis of Amplex Red to resorufin by HRP. (b) Resorufin emission intensity as a function of time for a ZIF-8 mesh that was exposed to ethanol and was not soaked in HRP solution (black, squares), a mesh that was not exposed to ethanol but was soaked with HRP (red circles), and a mesh that was exposed to ethanol and was soaked in HRP solution (blue triangles). All measurements were performed with meshes of similar fiber densities and with similar concentrations of reagents. (c) Similar experiment repeated with HKUST-1 meshes.

In a typical procedure, meshes of similar fiber densities containing the MOF precursors before and after exposure to ethanol were soaked in a solution containing an enzyme for a couple of hours. The meshes were then thoroughly washed in distilled water to remove the residual unbound enzyme. Next, the meshes were immersed in a solution containing the enzyme’s substrates—hydrogen peroxide and Amplex Red (AR). Throughout the catalysis process, AR is oxidized to the fluorophore resorufin (Figure 8a), and the fluorescence intensity of AR (Ex, 571 nm; Em, 585 nm) in the solution was monitored as a function of time to follow the reaction kinetics (Figures S9 and S10 in the Supporting Information).

Figure 8b depicts a comparison of the resorufin fluorescence intensity as a function of time for three different meshes containing ZIF-8 precursors: (a) a mesh that was exposed to ethanol and developed ZIF-8 crystals but was not immersed in the HRP solution, (b) a mesh that was not exposed to ethanol but was immersed in the HRP solution, and (c) a mesh that was exposed to ethanol (grew ZIF-8 crystals) and immersed in the HRP solution. All the meshes were exposed to similar concentrations of the substrates AR and hydrogen peroxide.

In the first system (Figure 8b, black squares), almost no catalytic activity was observed, indicating that the MOFs themselves do not act as catalysts for the reaction. The second system exhibited a moderate catalytic activity (Figure 8b, red circles), which can be attributed to enzymes that physisorbed to the surface of the polymeric fibers. However, in the third system, in which ZIF-8 crystals were grown, a significantly higher activity is reached, and the substrates were fully consumed after ∼5 min (Figure 8b, blue triangles). The catalysis rate was quadrupled with respect to the mesh that was not exposed to ethanol. We attribute the significantly higher activity to the improved immobilization of HRP to the ZIF-8 crystals.

Figure 8c shows a comparison of the fluorescence intensity of resorufin in the solution as a function of time for three different HKUST-1 meshes exposed to similar concentrations of the substrates AR and hydrogen peroxide. A similar trend to the ZIF-8 system is also seen here; however, the overall performance is lower. This reduced activity is attributed to the lower stability of HKUST-1 in the aqueous environment, which leads to its decomposition over time and, hence, to reduced enzyme immobilization.

In addition to HRP, we also examined the immobilization of the enzyme catalase to ZIF-8 meshes.^65−67^ Catalase decomposed hydrogen peroxide to water and oxygen, and hence, the performance can be qualitatively examined by the formation of oxygen bubbles on the mesh. Figure S10 shows photographs of ZIF-8 meshes exposed to the substrate hydrogen peroxide. In the case of meshes that were not immersed in a catalase solution, no oxygen bubbles were formed. A mesh that was not exposed to ethanol exhibited only a small amount of oxygen formation. The mesh with ZIF-8 crystals exhibited significantly higher formation of bubbles, indicating an improved immobilization of catalase. Furthermore, even a week after preparation, the MOF-containing meshes still exhibited high activity, suggesting that the MOFs also stabilized the immobilized enzymes. These results demonstrate the potential of the MOF meshes as durable and flexible solid scaffolds for enzyme immobilization and stabilization.

## Conclusions

In this article, we demonstrated a new synthetic route for the *in situ* growth of two types of MOFs on electrospun microfibers. The obtained fibers exhibit loading of more than 20% by weight of HKUST-1 crystals and 15% by weight of ZIF-8 crystals, which are strongly embedded inside the polymer matrix, and stay intact upon application of a mechanical load. In addition, the growth of the MOF crystals enhances the fiber mechanical strength, and the MOF fibers exhibit improved immobilization of enzymes, leading to the formation of meshes with high catalytic activity. The obtained results demonstrate the use of electrospun polymeric fibers as microreactors for performing chemical reactions that modify the properties of the fibers, lead to new functionalities, and pattern active material on top and inside the fiber. The scheme demonstrated here can be extended to hierarchical structures of various MOFs that could lead to performance fabrics with high potential for various applications, including filtering membranes and catalysis, in the near future.

## Experimental Section

### Materials

Polylactic-*co*-glycolic acid (PLGA) (lactide-to-glycolide ratio = 85:15; MW = 50–75 kDa), polyvinylpyrrolidone (PVP) (MW, ∼1,300,000), copper (II) nitrate trihydrate (Cu(NO_3_)_2_·3H_2_O; 99.9%), benzene-1,3,5-tricarboxylic acid (BTC) (≥98%), zinc acetate dihydrate (Zn(O_2_CCH_3_)_2_·2H_2_O; 99.0%), dimethyl sulfoxide (DMSO), tetrahydrofuran (THF), and dimethylformamide (DMF) were purchased from Sigma-Aldrich. Ethanol and methanol were purchased from BioLab. 2-Methylimidazole (Hmim) was purchased from Alfa Aesar. All the materials were used as bought without further purification.

### Jetting Solutions

For all the solutions described below, the concentrations are given in gram polymer to milliliter solvent. For the spinning of PLGA, a PLGA solution of 0.400 g/mL was prepared by dissolving 0.400 g of PLGA in a mixture of 0.5 mL of THF and 0.5 mL of DMF (1:1, v/v). The PVP/HKUST-1 precursor solution was prepared by dissolving 0.100 g of PVP, 0.280 g of copper nitrate trihydrate (1.16 M), and 0.129 g of BTC (0.614 M) in 1 mL of DMSO.^50^ The PVP/ZIF-8 precursor solution was prepared by dissolving 0.100 g of PVP, 0.280 g of zinc acetate dihydrate (1.27 M), and 0.210 g of Hmim (2.55 M) in 1 mL of DMSO.^51^

### Fiber Electrospinning

The experimental setup contained two syringe pumps (New Era), a power supply (DC voltage source, Gamma High Voltage Research, USA), and a rotating drum collector. The relative humidity in the room was between 50 and 65%. Above 70% humidity, the growth of the MOF crystals was significantly hindered, and no growth was observed.

The PLGA and PVP/precursor solutions were dispensed via a metallic coaxial core–shell needle made in-house using a 23-gauge needle and a 14-gauge needle. The PVP solution was dispensed at a constant flow rate of 0.200 mL·h^–1^, and the PLGA solution was also dispensed at a constant flow rate of 0.200 mL·h^–1^. A driving voltage of 1.5–2 kV resulted in a stable jet and the core–shell fibers were collected on a collecting drum at a tip-to-ground distance of 14 cm and a drum velocity of 80 rpm. Nonwoven meshes were formed by repeatedly moving the dispensing tip back and forth hundreds of times at a velocity of 0.1 cm per second.

### Instrumentation

Scanning electron microscopy (SEM) was performed using a Quanta 200FEG environmental scanning electron microscope in a high vacuum (WD, ∼10 mm; 3–20 kV).

Powder X-ray diffraction (PXRD) was performed using a Bruker D8 Advance diffractometer in parallel beam configuration, with a linear detector LYNXEYE_XE. The pattern was collected by 2θ scan between 4° and 60° with an omega angle fixed at 4° with CuK_α_ monochromatic radiation (λ = 1.5418 Å), operated at 40 kV and 40 mA. The scan speed was 1 s/step and the step size was 0.025°. Structural modeling was performed on the Cerius2 software suite.

Thermogravimetric analysis (TGA) was performed on a TGA Q5000 V3.17 Build 265 instrument in a nitrogen environment in a temperature range of 40–1000 °C (20 °C/min). TGA was carried out in a nitrogen atmosphere with a purge rate of 25 mL/min.

X-ray photoelectron spectroscopy (XPS) measurements were performed in UHV (2.5 × 10^–10^ Torr base pressure) using a 5600 Multi-Technique System (PHI, USA). The sample was irradiated with an AlK_α_ monochromated source (1486.6 eV) and the outcome electrons were analyzed by a spherical capacitor analyzer using the slit aperture of 0.8 mm. The samples were charged during measurements and a charge neutralizer was used for charge compensation.

Tensile measuements were performed using the Instron system with a load cell of maximum 100 N and the samples were pulled in a constant speed of 1 mm/min. LaVision’s Davis software was used to record and process the data.

Fluorescence measurement of resorufin for deducing the enzyme activity was performed using an Agilent Technologies Cary Eclipse fluorescence spectrometer. The excitation was performed at a wavelength of 550 nm, and emission was collected in the range of 560–650 nm (scan rate, 600 nm/min; averaging time, 0.1 s; excitation and emission slit openings, 2.5 nm; PMT detector voltage, 600 V).

### Enzyme Immobilization

Horse radish peroxidase (HRP) or catalase were adsorbed to the surface of meshes of ZIF-8 or HKUST-1 fibers before and after exposure to ethanol vapor. All of the ZIF-8 and the HKUST-1 meshes were of similar density.

Enzyme surface inmmobilization was performed for both the ZIF-8 and the HKUST-1 systems (for each system, before and after exposure to ethanol vapor) similarly. One milliliter of the enzyme in solutiom (1 mg/mL in phosphate buffer, pH 6) was added into a Petri dish. Meshes of ZIF-8 fibers or HKUST-1 (for each system, both before and after exposure to ethanol vapors) were added to the Petri dish and were left to gently stir at room temperature for a couple of hours. The meshes were then thoroughly washed with distilled water to remove unbound enzyme.

### HRP Activity Test

After the HRP immobilization, the ZIF-8 and HKUST-1 meshes were put into a solution containing the enzyme’s substrates—5 μL of Ampliflu Red (10 mM in DMSO), 5 μL of hydrogen peroxide solution (20 mM in water), and 5000 μL of phosphate buffer (pH 7). Upon catalysis, AR is oxidized by HRP in the presence of hydrogen peroxide to produce resorufin, a fluorescent compound whose concentration can be monitored through the fluorescence intensity (λ_ex_ = 571 nm and λ_em_ = 585 nm).

The activity of the catalase immobilized to the ZIF-8 and HKUST-1 systems was tested by adding the meshes into the substrate solution—1 mL of hydrogen peroxide solution (20 mM in water). Catalase decomposes hydrogen peroxide and oxygen bubbles are formed on the fibers.

## Supporting Information Available

Average diameters of the fibers and the crystals for HKUST-1 and ZIF-8 composite fibers, thermogravimetric analysis (TGA), MOF weight percentage in MOF composite fibers, a cross section of HKUST-1 fiber after exposure to ethanol, mechanically pulled ZIF-8 fiber SEM micrograph, SEM micrographs of a folded HKUST-1 fiber, tensile measurements of HKUST-1 fabrics, and catalase enzyme immobilization on MOF nonwoven meshes (PDF)
Mechanical deformation of nonwoven HKUST-1 fabric (Movie S1) (MP4)
