# Supplementary material for: Performance Fabrics Obtained by In Situ Growth of Metal–Organic Frameworks in Electrospun Fibers
Source: ACS Appl Mater Interfaces. 2021 Mar 4;13(10):12491–500. doi: 10.1021/acsami.0c22729 (PMC8034771; doi:10.1021/acsami.0c22729)
Supplement: Supplementary file 1 — am0c22729_si_001.pdf [file am0c22729_si_001.pdf]

Supporting information

Performance Fabrics Obtained by *In–situ* Growth of Metal-Organic Frameworks in Electrospun Fibers

Maya Molco[1], Fabrice Laye[2], Enrique Samperio[2], Shiran Ziv Sharabani[1], Victor Fourman[3], Dov Sherman[3], Manuel Tsotsalas,[2] Christof Wöll,[2] Joerg Lahann,[2] and Amit Sitt[1]*

1. School of Chemistry and the Tel-Aviv University Center for NanoScience and Nanotechnology, Tel Aviv University, Tel Aviv, 6997801, Israel

2. Institute of Functional Interfaces (IFG), Karlsruhe Institute of Technology (KIT), Eggenstein-Leopoldshafen, 76344, Germany

3. School of Mechanical Engineering, Tel-Aviv University, Tel-Aviv, 6997801, Israel

Corresponding Author

* Amit Sitt, School of Chemistry, Tel Aviv University, Tel Aviv, Israel, amitsitt@tauex.tau.ac.il

S1. Mean diameters of the fibers and the crystals for HKUST-1 and ZIF-8 composite fibers

The mean diameters of the fibers and of the MOF crystals were measured from SEM micrographs. To obtain the size distribution of the fibers, one hundred MOF composite fibers were measured for each MOF system before the exposure to ethanol vapor. The fiber diameter did not change significantly after the exposure to ethanol. To obtain the size distribution of the MOF crystals, the diameters of 150 crystals, embedded on top of the fibers, were measured. **Figure S1** presents the diameters distributions of the measured MOF composite fibers before the exposure to ethanol vapor, extracted from SEM images. Based on these measurements, the mean diameter of the HKUST-1 fibers is 19±4 μm, with average HKUST-1 crystal diameter 0.94±0.31 μm. For the ZIF-composite fibers, the average fiber diameter is 17±3 μm and the average ZIF-8 crystal diameter is 0.84±0.32 μm.

The diameter of the fibers is affected by environmental parameters such as humidity. **Figure S2** shows SEM images and diameter distribution of ZIF-8 fibers fabricated at low humidity levels

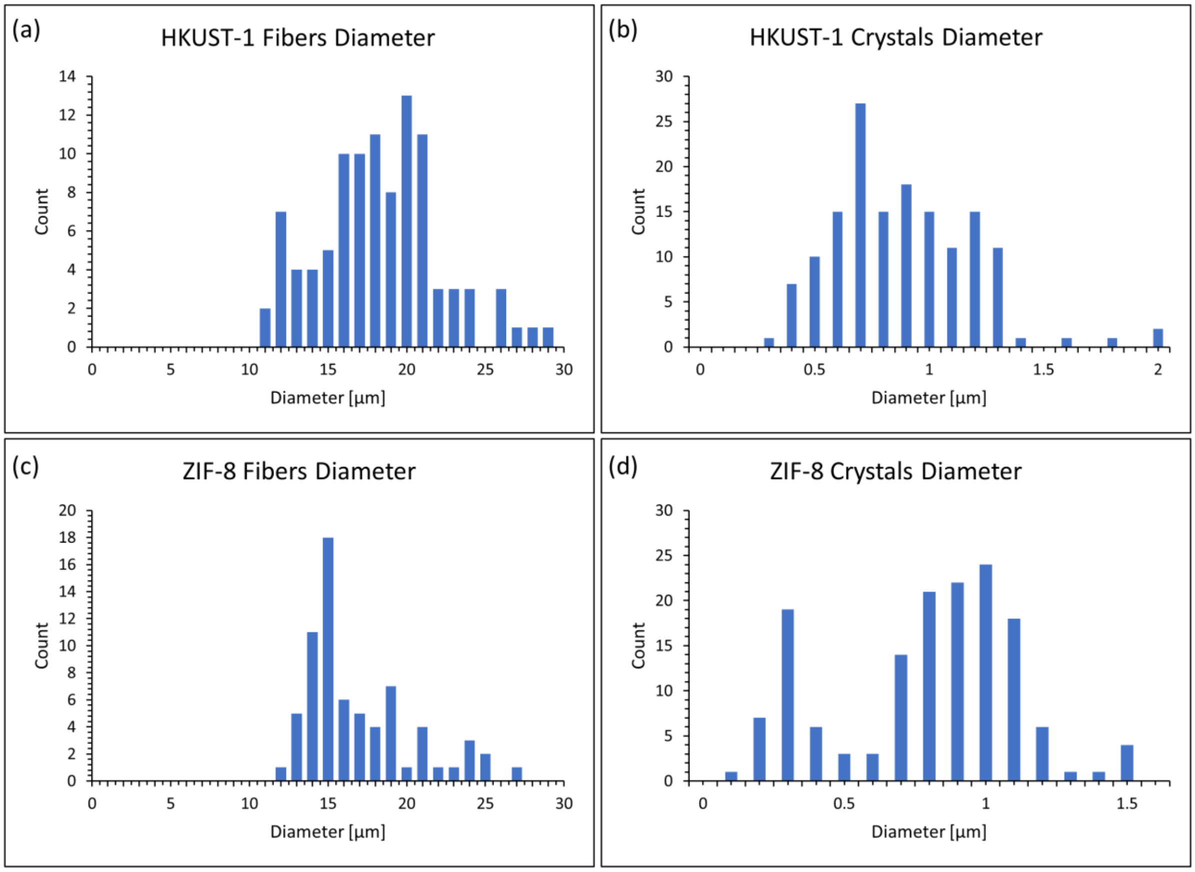

Figure S1: The diameter distribution of (a) the HKUST-1 fibers before the exposure to ethanol vapor and (b) the HKUST-1 crystals. The diameter distribution of (c) the ZIF-8 fibers before the exposure to ethanol vapor and (d) the ZIF-8 crystals.

(~35-45%). The mean diameter of these fibers is 10±3 μm, lower than the average diameter of the fibers fabricated at ~55-65% humidity (17±3 μm).

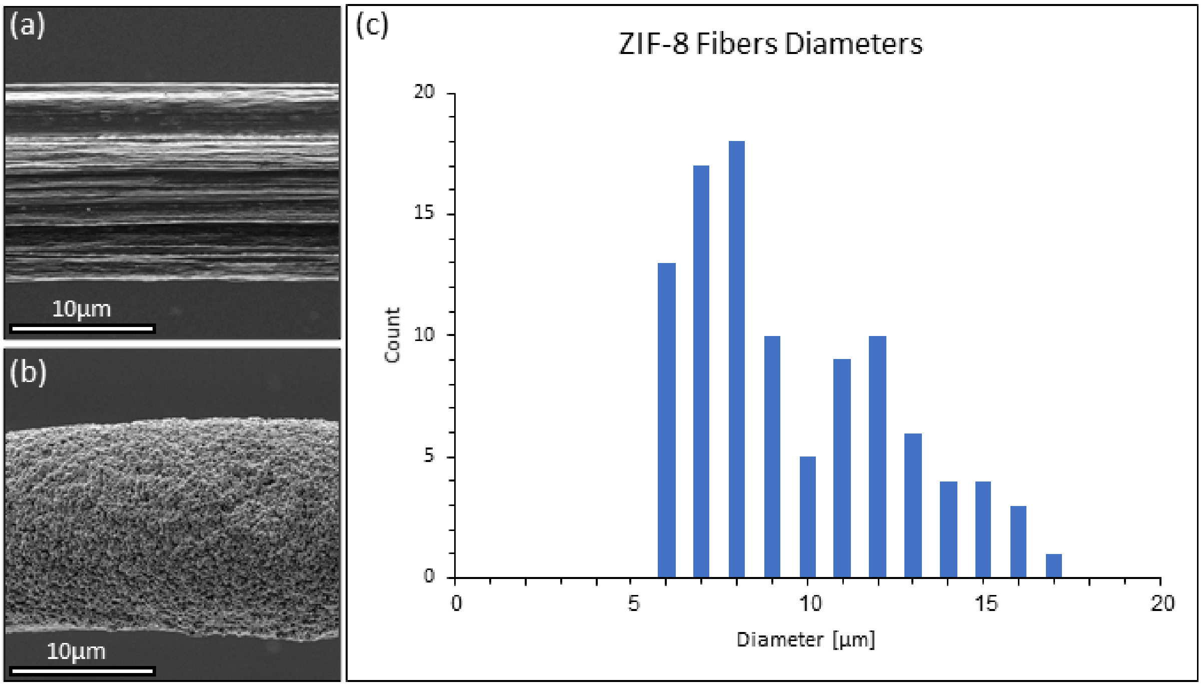

Figure S2: ZIF-8 fibers fabricated at low humidity levels (~35-45%). SEM micrographs of a single ZIF-8 fiber, fabricated at low humidity level, before (a) and after (b) the exposure to ethanol vapor. (c) Diameter distribution of the ZIF-8 fibers fabricated at low humidity level before the exposure to ethanol vapor.

S2. Thermogravimetric analysis (TGA)

TGA was performed on a mesh of MOF composite fibers after the exposure to ethanol vapor and on the pure components composing each of the systems under $N_{2(g)}$ atmosphere **(Figure S3)**. In the case of the pure polymers, no residues are left at the end of the measurement. Pure MOF samples, on the other hand, leave a residue of the metal oxides, which have much higher boiling temperatures. From the residue, we extract the weight percent of metal in our sample. This analysis indicated that the HKUST-1 fibers contain 8.7% copper by weight (10.87% copper oxide by weight as given by the TGA measurement). The total amount of copper added to the solution is 9.0%, indicating that the amount of copper in the fibers does not change during the jetting process. The TGA for the ZIF-8 fibers indicated 9.8% zinc by weight (12.24% zinc oxide by weight as measured in the TGA) while the total amount of zinc added to the solution was 8.4%. The slight deviation of the zinc percentage in the system can be attributed to the heterogeneity of the system.

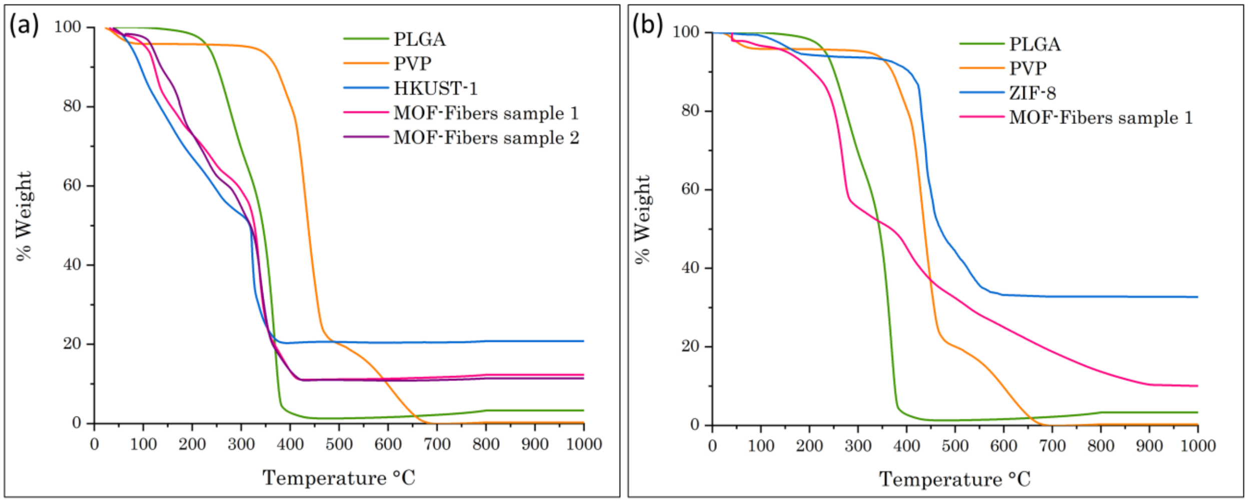

Figure S3: TGA curves for (a) HKUST-1 composite fibers and each of its components and for (b) ZIF-8 composite fibers and of each of its components. The pure polymers do not leave any residue, while the pure MOF leaves a residue of the metal oxides. The curves indicate the decomposition of the polymers and the crystals results in 10.87% copper oxide in the HKUST-1 system and 12.24% zinc oxide in the ZIF-8 system.

S3. MOF weight percentage in MOF composite fibers

The MOFs weight percentage in the MOF composite fibers was calculated in three different approaches: XRD, gravimetric analysis and TGA. The XRD and the gravimetric analysis for the HKUST-1 composite fibers indicates an almost similar mass percentage of the MOF in the system (23% and 22% respectively) with an average of 22% MOFs by weight. The yield of the process based on the XRD and gravimetric analysis is ~50% for the HKUST-1 system. The ZIF-8 system also showed a similar mass percentage according to the XRD and the gravimetric analysis (15% and 18% respectively) with an average of 16%. The yield of the process according to the first two methods is ~34%. As described above, the TGA overestimates the amount of MOF because it also accounts for unreacted metal. The summary of the mass percentage given by the different methods and the yields of the process in the two systems is shown in **table S1**.

	HKUST-1 fibers	ZIF-8 fibers
Based on precursor masses	45%	49%
XRD	23%	15%
Gravimetric analysis	22%	18%
Average	**22%**	**16%**
Yield	**50%**	**34%**
TGA	30%	34%

Table S1: Summary of the different quantitative analyses for the percentage of the MOF crystals in the samples.

The mass percentage according to the XRD was calculated by the following equation:

$$\%Amorphous = \frac{Global\ area - Reduced\ area}{Global\ area} \times 100\%$$

$$\%Crystalline = 100\% - \%Amorphous$$

The global area is the area underneath the whole XRD graph (taken automatically), and the reduced area is the area of the peaks (reducing the background, adjusted by the user).

S4. Cross section of HKUST-1 fiber after the exposure to ethanol

A cross section of HKUST-1 fiber after the crystal growth process was examined to further investigate the dispersion of the crystals in the fiber. After the exposure to ethanol vapor, HKUST-1 fibers were cooled using liquid nitrogen and cut using a sharp blade. **Figure S4** indicates that the crystals are positioned mainly on the fiber's surface, and no MOF crystal are visible inside the fiber, in accordance to figure 4 in the main text.

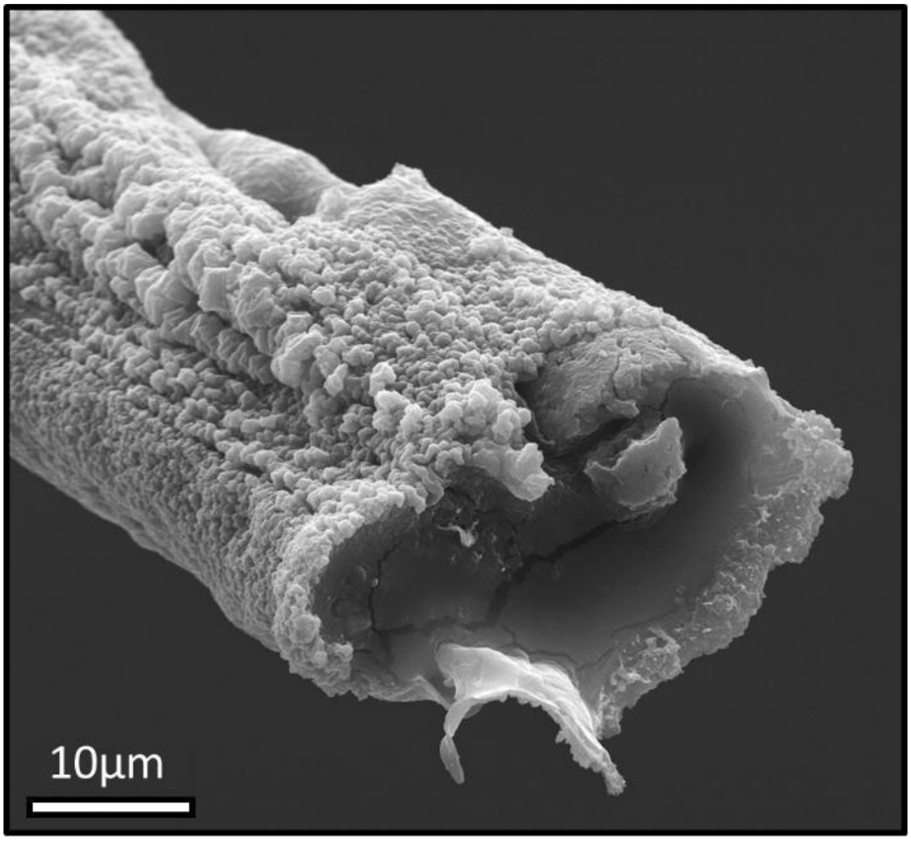

Figure S4: A SEM micrograph of a cross section of HKUST-1 fiber after the exposure to ethanol vapor. The crystals are covering the surface of the fiber but do not reside in its core.

S5. Mechanically pulled ZIF-8 fiber

A ZIF-8 composite fiber after the exposure to ethanol was mechanically pulled in the SEM. When pulled, some fibers exhibit fractures in the MOF layer, exposing the smooth polymer fiber underneath.

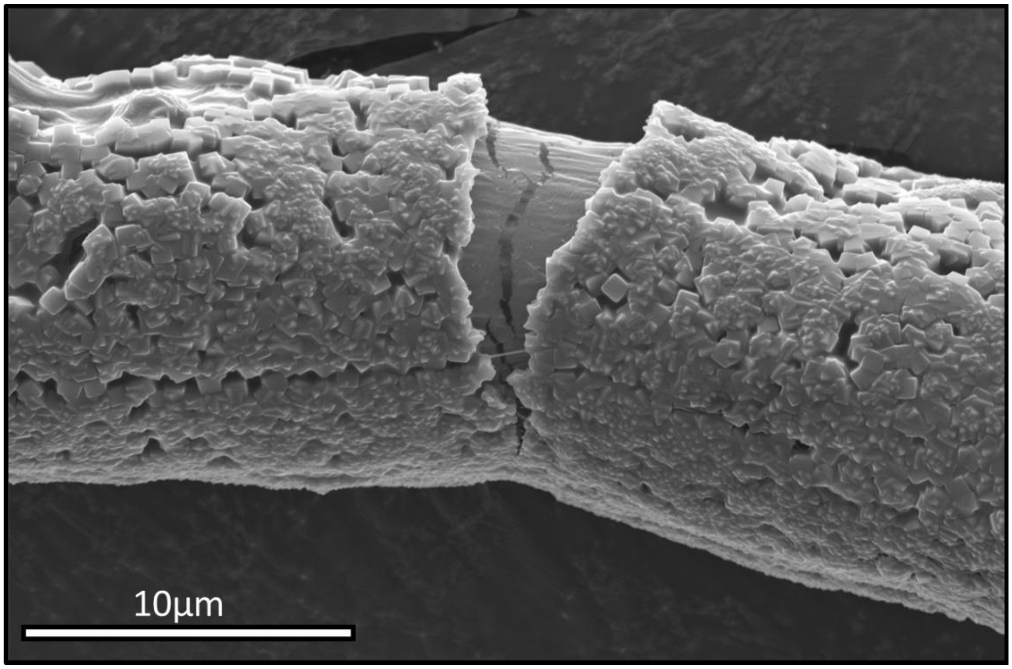

Figure S5: A SEM micrograph of a starched ZIF-8 composite fiber after the exposure to ethanol vapor. The MOF layer covering the fiber broke exposing the polymeric backbone of the fiber.

S6. Folding a HKUST-1 fiber

To confirm that the there is no loss of embedded MOF crystals after folding and twisting of the fibers, SEM micrographs of the same HKUST-1 fiber after exposure to ethanol vapor were taken before and after the application of mechanical load on the fiber. As can be seen in **Figure S6**, the mechanical load applied on the fiber did not affect the density of crystals covering the fiber or their consistency, thus, reinforcing the assumption that the crystals are strongly embedded into the fiber.

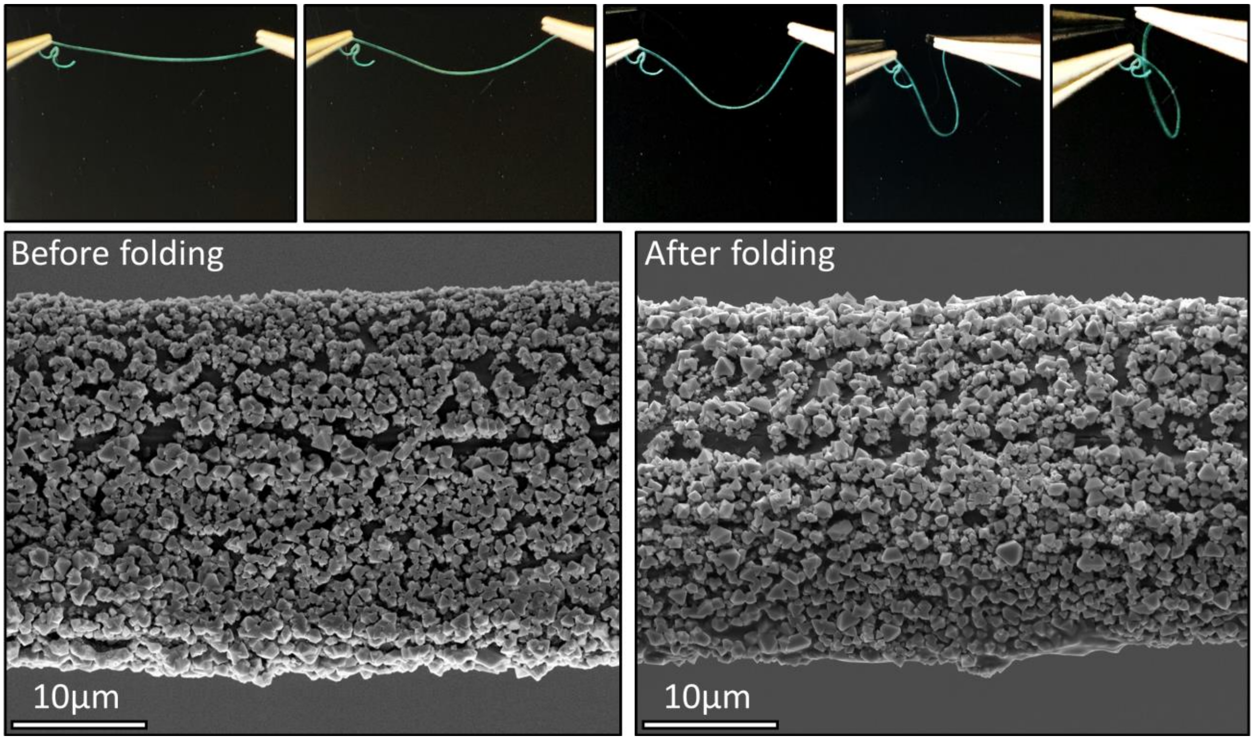

Figure S6: Top: A single HKUST-1 fiber held between two tweezers applying a mechanical stress. **Bottom:** SEM micrographs of the folded fiber before (left) and after (right) the folding. No significant loss of embedded crystals or deterioration of the MOF layer is observed.

S7. Tensile strength measurements of HKUST-1 fabric

Tensile strength measurements were performed on fabrics of fibers before and after the growth of the MOFs crystals to determine the effect of the growth on the mechanical properties of the fibers. For analyzing the mechanical properties of the fibers, a thick fabric with the dimensions of 12×30 cm^2 was electrospun on a rotating drum. The fabric was cut into two sections. Only one of the sections was exposed to ethanol vapor and MOF crystals were grown within the fibers. Each half fabric was cut into 14 strips of 2×4.5 cm^2, and each strip was glued using epoxy glue onto a cardboard holder (**Figure S7a**). The holder was placed in an Instron 5948 Universal Testing Machine equipped with a load cell of 100 N and its sides were cut so that the force will be applied only on the fabric (**Figure S7b**). The sample was then pulled in a constant speed of 1.00 mm/min until reaching the tensile strength (**Figure S7c.**).

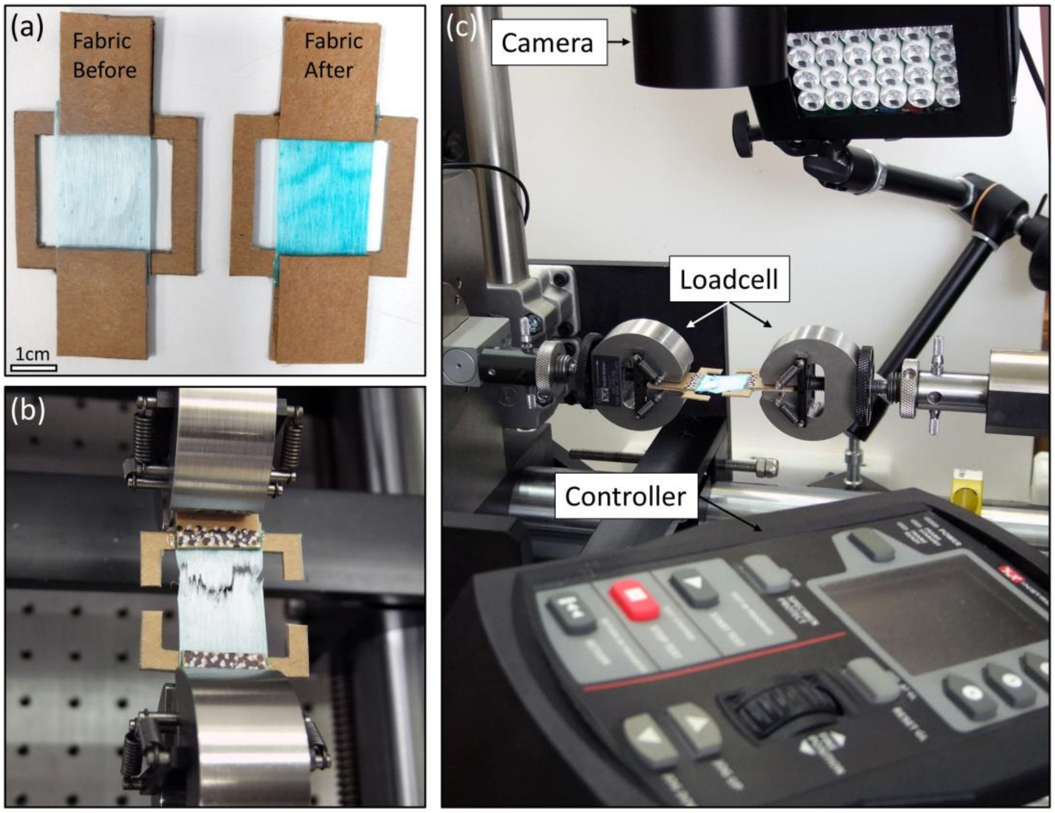

Figure S7: (a) a photo of the fabrics before (right) and after (left) the exposure to ethanol vapor glued to the cardboard holder. (b) A photo of the sample and the holder in the loadcell after the experiment. The cardboard holder is cut on the sides and the fabric is torn after being pulled. (c) A photo of the whole experimental setup including a camera for filming the process, the loadcell with the sample and the controller for adjusting the loadcell so that no force is applied on the sample before the measurement begins.

Figure S8a shows the tensile strength distribution graphs of the different samples, indicating on statistical distribution of the strength and strain. As can be clearly seen, the unexposed strips (Untreated, magenta) exhibit both a lower Young modulus (obtained from the slope at low strains) and a significant lower tensile strength than the exposed strips with grown MOFs (Treated, cyan). The differences between the samples are also clear when inspecting the tensile load histogram (**Figure S8c**), indicating that the maximal tensile loads of the untreated samples are all below 10 N, while all the treated samples exhibited tensile loads above 10 N. While in general, the strips that were exposed to the ethanol exhibited lower maximum strains, the untreated strips exhibited a larger range of strains (**Figure S8d**). The Young moduli of the treated samples are almost twice as large as the untreated samples, indicating the higher strength of the sample post growth.

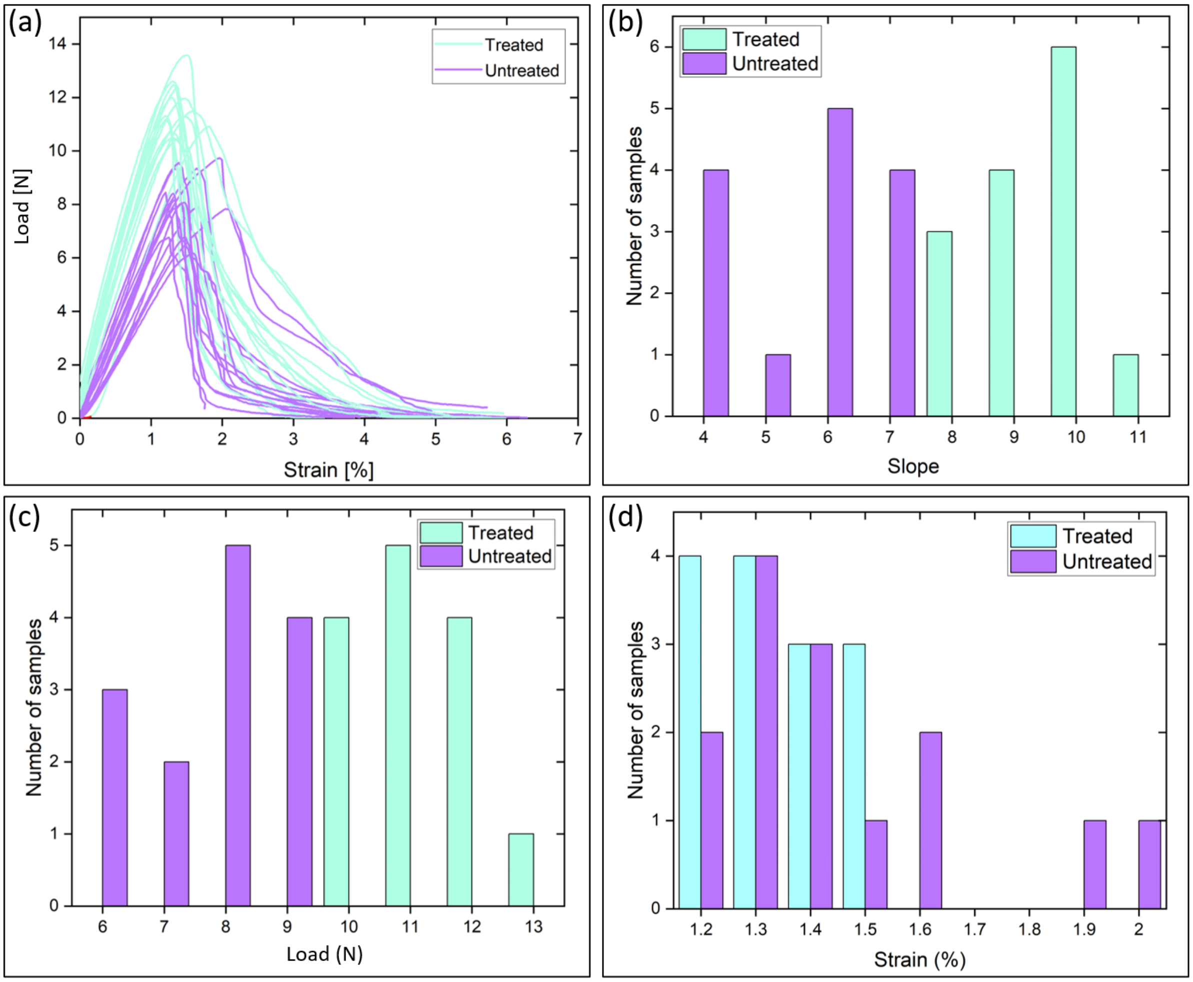

Figure S8: (a) a graph showing all the tensile measurement of the treated (cyan) and untreated (magenta) fabrics. Histograms of the slope (b) indicative for the Young modulus, the load (c), and the strain (d) of the treated (cyan) and the untreated (magenta) samples.

To examine the statistical significance of the difference in behavior, we performed a Weibull tensile strength distribution analysis of the samples. In this analysis, highly accepted for characterizing failure of brittle materials, the cumulative probability function is written such that the probability of the tensile failure, P_t, increases with the normalized fracture load L:[1, 2, 3]

$$P_t = 1 - \exp\left[-\left(\frac{L}{L_0}\right)^m\right]. \quad \text{S1}$$

where L_0 is the characteristic (mean) load, and *m* is the Weibull modulus.

Taking the double logarithm of the resulting two-parameter Weibull distribution yields:

$$\ln\left(\ln\left(\frac{1}{1-P_t}\right)\right) = m(lnL - lnL_0) \quad \text{S2}$$

Defining $S = 1 - P_t$, **Figure S9** shows the fitting of the data obtained for the treated and untreated strips with equation S2. The fit captures the behavior of the systems, and indeed both the characteristic strengths and the Weibull moduli of the samples before and after exposure are significantly different. The modulus of the treated samples is more than double that of the untreated samples, indicating a significant increase in the fiber strength after the exposure and growth of the MOF crystals.

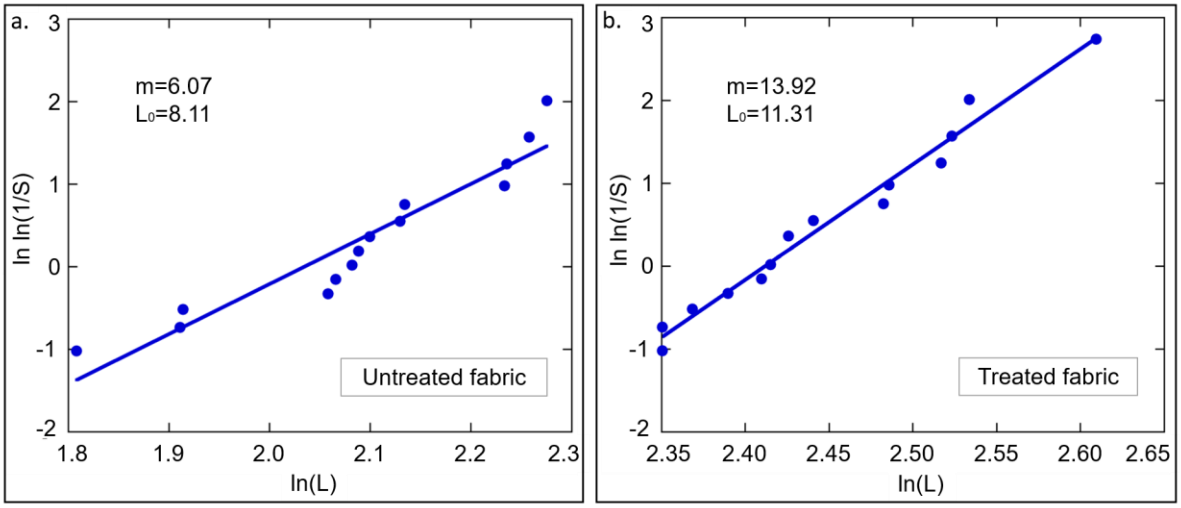

Figure S9: (a) Weibull statistics graph of 14 untreated HKUST-1 fabric samples (before the exposure to ethanol). (b) Weibull statistics of 14 HKUST-1 treated fabric samples (after the exposure to ethanol). The two types of samples (treated and untreated) have a significantly different Weibull modulus (m) and mean load at which the fabric is torn (L_0).

S8. Fluorescence of resorufin as function of time for different meshes

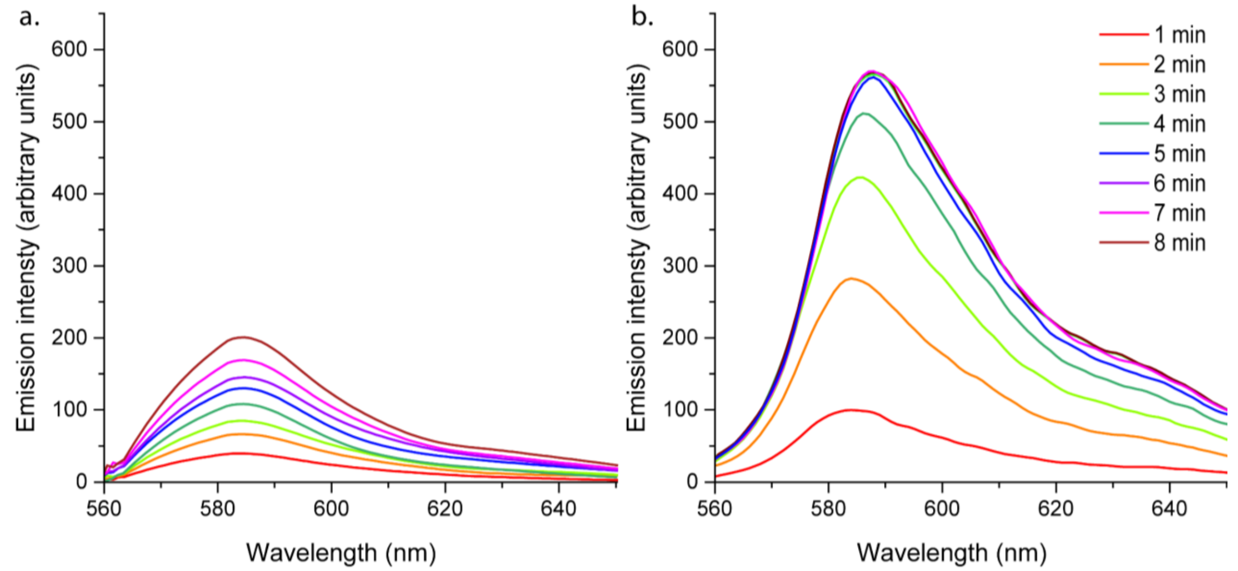

Figure S9: Fluorescence of resorufin excited at a wavelength of 550 nm as function of time for meshes containing ZIF-8 precursors that were immersed in HRP solution: (a) a mesh before exposure to ethanol, and (b) a mesh after the exposure to ethanol and growth of ZIF-8 MOF on the fibers. The maximum emission intensity was used to construct Figure 8b.

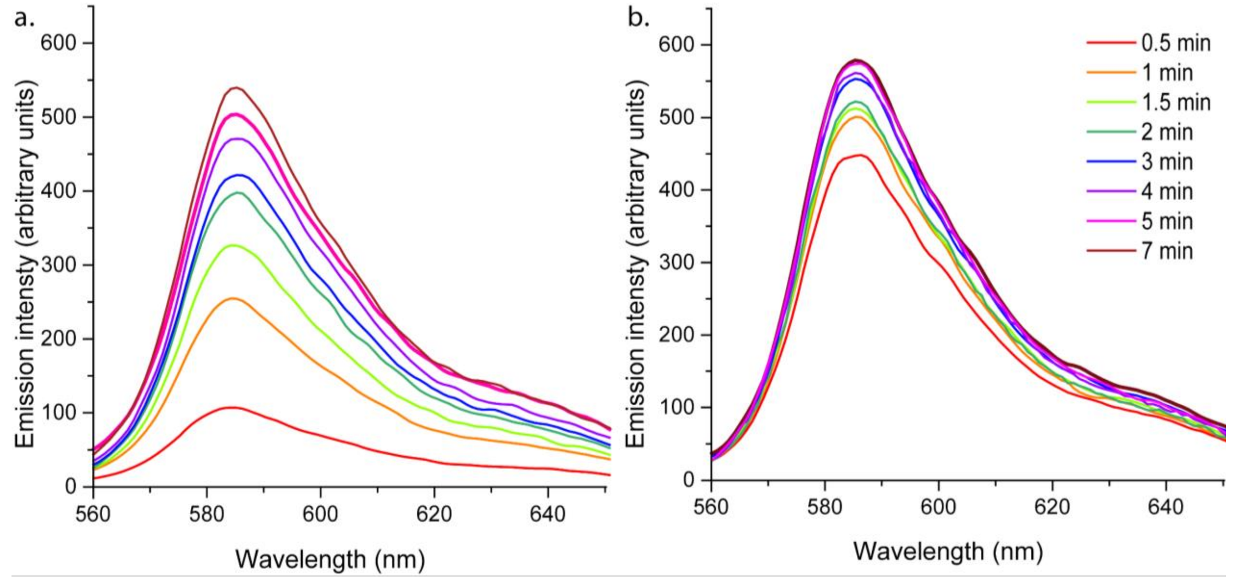

Figure S10: Fluorescence of resorufin excited at a wavelength of 550 nm as function of time for meshes containing HKUST-1 precursors that were immersed in HRP solution: (a) a mesh before exposure to ethanol, and (b) a mesh after the exposure to ethanol and growth of HKUST-1 MOF on the fibers. The maximum emission intensity was used to construct Figure 8c.

S9. Immobilization of catalase on meshes with MOF-precursors

The immobilization of catalase on ZIF-8 MOF fibers was examined on three meshes of similar fiber density: (a) a mesh that was exposed to ethanol and developed ZIF-8 crystals, but was not immersed in the catalase solution, (b) a mesh that was not exposed to ethanol but was immersed in the catalase solution for two hours, and (c) a mesh that was exposed to ethanol (grew ZIF-8

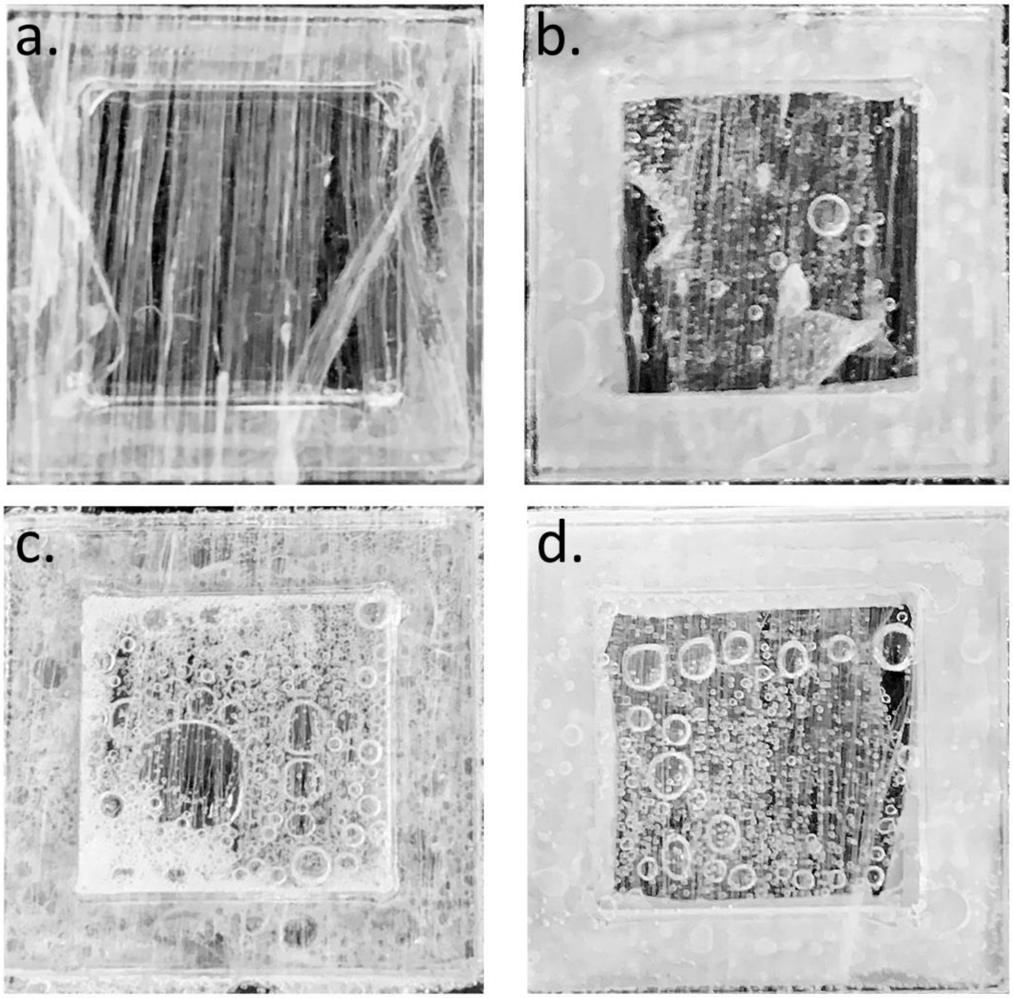

Figure S11: Catalytic activity of meshes containing ZIF-8 precursors stretched over a poly methyl methacrylate frame (1cm×1cm) immobilized with catalase. Oxygen bubbles formed on the mesh indicate the catalytic decomposition of H_2O_2 to oxygen: (a) A mesh after exposure to ethanol and growth of ZIF-8 MOFs, which was not immersed in a catalase solution. (b) A mesh that was not exposure to ethanol which was immersed in a catalase solution. (c) A mesh that after exposure to ethanol and MOF-growth, which was immersed in a catalase solution. (d) The same mesh after one week.

crystals) and was immersed in the catalase solution for two hours. All the meshes were immersed in a solution of 20mM in water immersed in the catalase solution. The meshes that were immersed in the catalase solution were thoroughly washed to remove unbound catalase.

Figure S11 shows the meshes (which were stretched over a frame poly methyl methacrylate frame for easier handling) after the addition of 20 mM H_2O_2 in water. The mesh which was not immersed in the catalase solution (Figure S11 (a)) did not show any activity. The mesh that was not exposed to ethanol but was immersed in the catalase solution (Figure S11 (b)) showed some activity, probably because of the physisorption of catalase to the polymer fibers. However, the mesh that was exposed to ethanol and has ZIF-8 MOFs on his surface (Figure S11 (c)), exhibited significantly higher activity, indicating improvement in the immobilization of the enzyme due to the formation of the MOF.

After the experiment, the meshes were washed and dried, and their performance was re-examined after one week. Only the mesh containing the ZIF-8 MOF exhibited activity after a week (Figure S11(d)), indicating that in addition to improved immobilization, the MOFs also significantly improve the stability of the enzyme.

References:

1. Quinn, J. B., & Quinn, G. D., A practical and systematic review of Weibull statistics for reporting strengths of dental materials. Dental materials **2010**., 26, 135–147.

2. Davidge, R. W., Mechanical Behaviour of Cereamics, Cambridge University Press, London, **1979**.

3. Weibull, W. *The phenomenon of rupture in solids*. Generalstabens litografiska anstalts förlag, Stockholm **1939**.
